# A Data Collection Strategy for Heterogeneous Wireless Sensor Networks Based on Energy Efficiency and Collaborative Optimization

**DOI:** 10.1155/2021/9808449

**Published:** 2021-09-29

**Authors:** Li Cao, Yinggao Yue, Yong Zhang

**Affiliations:** ^1^School of Intelligent Manufacturing and Electronic Engineering, Wenzhou University of Technology, Wenzhou 325035, China; ^2^Computer School, Hubei University of Arts and Science, Xiangyang 441053, China

## Abstract

In the clustering routing protocol, prolonging the lifetime of the sensor network depends to a large extent on the rationality of the cluster head node selection. The selection of cluster heads for heterogeneous wireless sensor networks (HWSNs) does not consider the remaining energy of the current nodes and the distribution of nodes, which leads to an imbalance of network energy consumption. A strategy for selecting cluster heads of HWSNs based on the improved sparrow search algorithm- (ISSA-) optimized self-organizing maps (SOM) is proposed. In the stage of cluster head selection, the proposed algorithm establishes a competitive neural network model at the base station and takes the nodes of the competing cluster heads as the input vector. Each input vector includes three elements: the remaining energy of the node, the distance from the node to the base station, and the number of neighbor nodes of the node. The best cluster head is selected through the adaptive learning of the improved competitive neural network. When selecting the cluster head node, comprehensively consider the remaining energy, the distance, and the number of times the node becomes a cluster head and optimize the cluster head node selection strategy to extend the network life cycle. Simulation experiments show that the new algorithm can reduce the energy consumption of the network more effectively than the basic competitive neural network and other algorithms, balance the energy consumption of the network, and further prolong the lifetime of the sensor network.

## 1. Introduction

Over the years, the technology of heterogeneous wireless sensor networks (HWSNs) has been developed by leaps and bounds. Using its own advantages, it has been widely used in environmental detection, smart home, public transportation, and other fields [1]. However, due to its own software and hardware conditions, the storage, computing, and communication capabilities are weak; especially, the energy is limited and cannot be recharged in real time. Therefore, traditional routing protocols are not suitable for heterogeneous wireless sensor networks [2, 3]. How to balance energy consumption and prolong the lifetime of the network has become the focus and difficulty of designing routing protocols for heterogeneous wireless sensor networks [4–6].

The computing power, storage capacity, and power of the sensor nodes of heterogeneous wireless sensor networks are also very limited [7]. Therefore, reducing node energy consumption and cost is one of the main considerations in the design of heterogeneous wireless sensor networks. A large number of research results continue to show that it has practical theoretical and practical significance to propose innovative routing algorithms and effectively use available energy to ensure that the network is more robust, reliable, and accurate [8, 9]. The routing in heterogeneous wireless sensor networks is divided into plane routing, hierarchical routing, and location-based routing which depends on the network structure. Hierarchical routing is also called cluster routing. Sensor nodes of this type of routing are grouped together and form clusters. In each cluster, the node with the higher energy is allocated as a cluster head node. Becoming a leader is responsibility for collecting and summarizing data by themselves, and the respective clusters and transmissions summarize the data in their respective clusters and forward them to the base station (BS) [10].

In a cluster, the closer the geographic location of the cluster head is to the center of the cluster, the smaller the average energy consumption of each node in the cluster is and the less energy consumption of the cluster head is [3]. Therefore, when selecting the cluster head, the remaining energy of the node and its average energy consumption per round are considered at the same time so that the greater the remaining energy and the smaller the average energy consumption per round, more chances the nodes have to become cluster heads. It can effectively prolong the life time of the sensor network [11].

A cluster head selection strategy for HWSNs based on the improved sparrow search algorithm (SSA) optimization self-organizing maps (SOM) is proposed in this paper. The basic idea is the proposed improved sparrow search algorithm optimizes the clustering routing algorithm of heterogeneous wireless sensor networks that compete with neural networks. In the cluster head selection stage, the improved sparrow search algorithm is used to optimize the principle of competitive neural network to select the best cluster head. The specific implementation process is to establish an improved competitive neural network in the base station and select the most suitable member head after the input vector of the sensor node of each cluster leader is processed through the input vector of the neural network layers [12, 13]. And, considering the establishment of a selection function and intermediate nodes for data forwarding, the collected data are finally forwarded to Sink. The proposed algorithm effectively reduces network energy consumption, balances network energy consumption, and prolongs the life cycle of the network.

In this work, a data collection strategy for heterogeneous wireless sensor networks based on energy efficiency and collaborative optimization is proposed. In comparison with the current general selection approaches, the main contributions of our work in this paper can be summarized as follows:

1. Characterize the issues of the data collection for HWSNs, and classify the current data collection of HWSNs
2. Propose a data collection method of heterogeneous wireless sensor network based on the improved sparrow search algorithm (ISSA) optimized self-organizing maps (SOM) (ISSA-SOM)
3. Evaluate the performance of the proposed algorithms by comparing them with the data collection methods of the PSO-SOM, GWO-SOM, and SSA-SOM algorithms

## 2. Related Work

Aiming at the characteristics of heterogeneous wireless sensor networks, the LEACH algorithm is to let each node take turns to act as the cluster head, so as to make the energy consumption in the network as even as possible and reduce the energy waste when the network fails. The LEACH algorithm is a very big breakthrough compared to flat networks, from traditional flat routing to hierarchical routing. However, this algorithm only considers the single-hop situation and does not consider the remaining energy of the node in the process of selecting the cluster head. The cluster head far away from the sink of the base station is easy to die. Although it has a great improvement over the flat network, it still has shortcomings.

The clustering routing protocol has greater advantages compared with the flat routing protocol; it has become a research hotspot in recent years. In [14], the authors discussed a cluster head weight selection method, called cluster chain weight metrics approach (CCWM), which uses service parameters to improve the performance of the entire network. In the clustering-based method, the appropriate cluster head is selected in the sensor network to balance the energy consumption of the network. In [15], the author proposed a cluster head selection method based on genetic algorithm, which defines a cost function on the basis of residual energy and distance parameters so that nodes near the node select cluster heads in a cluster, so as to achieve an ideal cluster distribution. This method can effectively reduce energy consumption, improve energy efficiency, and prolong the network's lifetime. In [16], the author proposed an improved LEACH-based cluster head selection algorithm (LEACH-M) for the disadvantages of unreasonable cluster head selection and excessive energy consumption in wireless sensor networks. Comprehensively, consider the remaining energy and network address of the node and optimize the cluster head selection strategy. In [17], the author proposed a hybrid grouped gray wolf search optimization algorithm (GGWSO) to improve the performance of cluster head selection in WSN, thereby prolonging the lifetime of the network. This method considers the main constraints of distance, delay, energy, and safety. How to deal with sensor nodes in an energy-saving manner is a difficult task. In order to solve this problem, the author of [18] proposed a cluster head selection method combining Nash equilibrium decision-making based on game theory, which effectively reduces network energy consumption and prolongs the life of the network. Choosing a cluster head can properly balance the load in the network, reduce energy consumption, and increase network life. In [19], the author studied an efficient cluster head selection scheme, which rotates the cluster head position among nodes with higher energy levels. The algorithm considers the initial energy, remaining energy, and optimal value of the cluster head and selects the appropriate cluster head node for the network suitable for IoT applications (such as environmental monitoring, smart cities, and systems).

With the rise of intelligent optimization algorithms, the combination of cluster head selection with neural networks and intelligent optimization algorithms has become closer and closer, which has attracted the attention of many experts and scholars. The author proposes an energy-aware clustering mechanism based on mobile sinks, which uses a firefly optimization algorithm inspired by nature to select cluster heads. Among them, the following parameters, remaining energy, average node-to-node distance, and node-to-sink distance are the decisive parameters of the cluster head selection process. This method overcomes the problem of energy holes and improves the survival time of the network [20]. In the cluster head selection, five different parameters are considered: distance node (DistNode), remaining energy level (REL), distance centroid (DistCent), number of times a node is selected as a cluster head (TCH), and merge node (MN). The cluster head selection problem based on these parameters is treated as a multicriteria decision system, and the analytical network process (ANP) method is used to optimize the selection of cluster heads [21]. In [22], the author proposed a cluster head selection strategy for HWSNs based on particle swarm optimization algorithm. This method selects the optimal cluster head better, reduces the network lifetime, and prolongs the network's lifetime. Aiming at the problem of cluster head selection in mobile heterogeneous wireless sensor networks, Verma et al. proposed a genetic algorithm-based cluster head selection method, which balances the energy consumption and improves the efficiency of the network [23]. In [24], the author proposed a fuzzy TOPSIS cluster head selection method based on multicriteria decision-making, which effectively selects the channel and maximizes the lifetime of the wireless sensor network. The comprehensive consideration of cluster head selection includes remaining energy, node energy consumption rate, number of adjacent nodes, average distance between adjacent nodes, and distance to the receiver. A threshold-based multihop communication mechanism within and between clusters is proposed to reduce energy consumption. Subramanian et al. proposed a cluster head selection strategy based on gray wolf optimization algorithm for the problem of increased energy consumption and increased transmission delay caused by the selection of cluster heads in heterogeneous sensor networks. This method effectively reduces network energy consumption and prolongs the network's lifetime [25].

This paper proposes a cluster head selection strategy for HWSNs based on the improved sparrow search algorithm (SSA) optimization self-organizing maps (SOM). An improved competitive neural network is established at the base station, and the sensor node input vector of each cluster leader is selected, and the most suitable leader is selected after processing the input vector through each layer of the neural network. When transferring data between the cluster member, considering the establishment of a selection function and intermediate nodes for data forwarding, the collected data are finally forwarded to Sink. The proposed algorithm effectively reduces network energy consumption, balances network energy consumption, and prolongs the lifetime of the network.

## 3. Sparrow Search Algorithm

Sparrow search algorithm (SSA), equivalent to the Sparrow Search Algorithm (SSA) proposed in 2020 by XueJiankai, is a new type of swarm intelligence optimization algorithm [26]. Compared with other swarm intelligence optimization algorithms, the SSA algorithm has the characteristics of high search accuracy, fast convergence speed, good stability, and strong robustness. The application of sparrow search algorithm has a wider range of scenarios, and it has a wide range of applications in function optimization, combinatorial optimization problems, NP-hard problems, path planning, image processing, and so on. However, the SSA algorithm is the same as other swarm intelligence optimization algorithms, when its search is close to the global optimum, there will still be problems such as reduced population diversity and easy to fall into local optimum [27].

The SSA algorithm is a new type of swarm intelligence optimization algorithm inspired by sparrow foraging behavior and antipredation behavior. Its bionic principle is as follows. In the SSA algorithm, the foraging process of sparrows is simulated to obtain a solution to the optimization problem [28]. Assuming that there are N sparrows in a *D*-dimensional search space, the position of the *i*th sparrow in the *D*-dimensional search space is *X*_*i*_ = [*x*_*i*1_,…, *x*_*id*_,…, *x*_*iD*_], where *i* = 1, 2,…, *N*. The parameter *x*_*id*_ represents the position of the *i*th sparrow in the *d*th dimension. Discoverers generally account for 10% to 20% of the population, and the location update formula is as follows:

(1) $$\begin{array}{c} x_{id}^{t+1}=\left\{\begin{array}{c} x_{id}^{t}\times \;\exp\left(\frac{-i}{\alpha \times T}\right),\;R_{2}<\text{ST}\\ x_{id}^{t}+Q\times L,\;R_{2}\geq \text{ST} \end{array}\right), \end{array}$$

where the parameter *t* represents the current number of iterations, *T* is the maximum number of iterations, and the parameter *α* is a uniform random number between (0, 1]. The parameter *Q* is a random number that obeys the standard normal distribution. The parameter *L* indicates that the elements are all the matrix of 1. The parameters *R*_2_ and ST represent the warning value and the safety value [29]. When *R*_2_ < ST, the population does not find the presence of predators or other dangers, the search environment is safe, and the discoverer can search extensively to guide the population to obtain higher fitness. When *R*_2_ ≥ ST, the sparrow detects the predator and immediately releases a danger signal. The population immediately performs antipredation behavior, adjusts the search strategy, and quickly moves closer to the safe area. Except for the discoverer, the remaining sparrows are all joiners and update their positions according to the following formula:

(2) $$\begin{array}{c} x_{id}^{t+1}=\left\{\begin{array}{c} Q\times \;\exp\left(\frac{xw_{d}^{t}-x_{id}^{t}}{i^{2}}\right),\;i>\frac{n}{2}\\ x\times b_{d}^{t+1}+\left|x_{id}^{t}-x\times b_{d}^{t+1}\right|\times A^{-},\;\text{otherwise} \end{array}\right). \end{array}$$

Formula (2) has a slight deviation, and it is modified as follows:

(3) $$\begin{array}{c} x_{id}^{t+1}=\left\{\begin{array}{c} Q\times \;\exp\left(\frac{xw_{d}^{t}-x_{id}^{t}}{i^{2}}\right),\;i>\frac{n}{2}\\ x\times b_{d}^{t+1}+\frac{1}{D}\sum_{d=1}^{D}\left(\text{rand}\left\{-1,1\right\}\times \left|x_{id}^{t}-xb_{d}^{t+1}\right|\right),\;i\leq \frac{n}{2} \end{array}\right). \end{array}$$

In formula (3), the parameter *xw*_*d*_^*t*^ represents the worst position of the sparrow in the *d*th dimension at the *t*th iteration of the population. The parameter *xb*_*d*_^*t*+1^ represents the optimal position of the sparrow in the *d*th dimension during the *t* + 1 iteration of the population. When *i* > *n*/2, it indicates that the *i*th joiner has no food, is hungry, and has low fitness. In order to obtain higher energy, you need to fly to other places for food. When *i* ≤ *n*/2, the *i*th joiner will randomly find a location near the current optimal position *xb* for foraging.

Sparrows for reconnaissance and early warning generally account for 10% to 20% of the population, and their positions are updated as follows [30]:

(4) $$\begin{array}{c} x_{id}^{t+1}=\left\{\begin{array}{c} x\times b_{d}^{t}+\beta \times \left(x_{id}^{t}-x\times b_{d}^{t}\right),\;f_{i}\neq f_{g}\\ x_{id}^{t}+K\times \left(\frac{x_{id}^{t}-x\times w_{d}^{t}}{\left|f_{i}-f_{w}\right|+e}\right),\;f_{i}=f_{g} \end{array}\right). \end{array}$$

In formula (4), the parameter *β* represents the step length control parameter, which is a normally distributed random number with a mean value of 0 and a variance of 1. The parameter *K* is a random number between [−1, 1], which represents the direction in which the sparrow moves and is also a step length control parameter. The parameter *e* is a very small constant to avoid the situation, where the denominator is 0. The parameter *f*_*i*_ represents the fitness value of the *i*th sparrow, and *f*_*g*_ and *f*_*w*_ are the best and worst fitness values of the current sparrow population, respectively. When *f*_*i*_ ≠ *f*_*g*_, it indicates that the sparrow is on the edge of the population and is vulnerable to attack by predators. When *f*_*i*_ = *f*_*g*_, it indicates that the sparrow is in the middle of the population. As it is aware of the threat of predators, in order to avoid being attacked by predators, it is necessary to approach other sparrows in time to adjust the search strategy.

## 4. SOM Optimized by Improved Sparrow Search Algorithm

The SOM neural network is a kind of neural network with strong self-supervision and self-learning ability [31]. It can extract data features from sample data and requires less sample size and can cluster data well. The basic idea of the competitive neural network is to allow each neuron in the competition layer to compete for the corresponding opportunity to the input mode. In the end, only one neuron becomes the winner of the competition. The output of this winning neuron represents the classification of the input pattern [32].

The learning algorithm steps are as follows:

(1) The random number initializes the weight *w*_*ij*_ between the input layer and the mapping layer.
(2) Input the vector to the input layer *X*=(*X*_1_, *X*_2_, *X*_3_,…, *X*_*n*_).
(3) Calculate the Euclidean distance *d*_*j*_ between the weight vector of each neuron and the input vector:
  (5) $$\begin{array}{c} d_{j}=\sqrt{\sum_{i=1}^{n}{\left(x_{i}-w_{ij}\right)}^{2}}. \end{array}$$
(4) Choose the neuron with the smallest as the winning neuron.
(5) Update the weights of the winning neuron and its neighborhood:
  (6) $$\begin{array}{c} \Delta w_{ij}=w_{ij}\left(t+1\right)-w_{ij}=\eta \left(t\right)\left(x_{i}-w_{ij}\right). \end{array}$$
(6) If the target requirement is reached, the algorithm ends. Otherwise, it returns to step (2).

Although the self-organizing competitive neural network algorithm is an unsupervised light intensity and other environmental factors, it is more efficient and accurate. The practical artificial intelligence algorithm has strong autonomous learning and environmental adaptability [33, 34]. However, it is still the same as other neural networks, and it needs to make a compromise between the learning rate and the stability of the final weight vector, and the initialization of the weights will affect the final training effect of the entire network. In some special cases, it may also lead to the emergence of “dead” neurons, thereby reducing the convergence speed and clustering accuracy of the network calculation. Therefore, the SOM network has a greater dependence on the setting of the initial weight, which will affect the convergence speed and learning effect of the network [35, 36].

As a simple and effective evolutionary algorithm, the improved SSA algorithm has strong global search capabilities and robustness and can effectively solve more complex optimization problems without the need for the feature information of the problem. In this paper, ISSA algorithm is used to solve the optimization problem of self-organizing competitive neural network so that the advantages of the two can be combined, and the original unsupervised learning becomes supervised learning so that the learning efficiency and accuracy of the network can be greatly improved. The improved sparrow search algorithm (ISSA) is used to optimize the setting of the initial weights of the competitive neural network SOM.

The SSA algorithm can achieve better optimization performance than PSO, GWO, and other algorithms, but its running time is longer, and it still has the defect of falling into local optimum. Therefore, combined with the idea of bird swarm algorithm (BSA), an improved sparrow search algorithm (ISSA) is designed to shorten the running time of the algorithm [37]. And, improve its search ability and development ability, and achieve better global optimization performance.

The BSA algorithm is a swarm intelligence algorithm proposed by Xian-Bing Meng et al., in 2015, inspired by bird flock flying, foraging, and alert behavior and has good stability [38]. The position update formulas of the discoverer and the joiner in the flight behavior are, respectively,

(7) $$\begin{array}{c} x_{ij}^{t+1}=x_{ij}^{t}+x_{ij}^{t}\times \text{rand}n\left(0,1\right), \end{array}$$

(8) $$\begin{array}{c} x_{ij}^{t+1}=x_{ij}^{t}+\left(x_{kj}^{t}-x_{ij}^{t}\right)\times \text{FL}\times \text{rand}\left(0,1\right),\;k\neq i. \end{array}$$

In the formula, rand*n*(0, 1) represents a Gaussian random distribution with a mean of 0 and a standard deviation of 1. The parameter FL∈[0, 2] represents the probability that the joiner follows the producer to find food [39]. Since the discoverer in the SSA algorithm is *R*_2_ < ST, each dimension of the sparrow individual is getting smaller, which is inferior to the search strategy of the discoverer in the flight behavior in the BSA algorithm [40].

Combined with the idea of bird swarm algorithm (BSA), an improved sparrow search algorithm (ISSA) is designed to shorten the running time of the algorithm, improve its search ability and development ability, and achieve better global optimization performance. Therefore, the update formula for the location of the discoverer in the SSA algorithm is improved as follows:

(9) $$\begin{array}{c} x_{ij}^{t+1}=\left\{\begin{array}{c} x_{ij}^{t}+x_{ij}^{t}\times \text{rand}n\left(0,1\right),\;R_{2}<\text{ST}\\ x_{id}^{t}+Q,\;R_{2}\geq \text{ST} \end{array}\right). \end{array}$$

At the same time, when the joiner in the SSA algorithm approaches the best position in all dimensions, although it can achieve the effect of rapid convergence, it reduces the diversity of the population, and the algorithm is easy to fall into the local optimum. The joiner in the BSA algorithm approaches the discoverer with a certain probability, which not only ensures global convergence but also does not lose the diversity of the population, effectively jumping out of the local optimum. Therefore, the position update formula of the joiner in the improved SSA algorithm is as follows:

(10) $$\begin{array}{c} x_{ij}^{t+1}=\left\{\begin{array}{c} Q\times \;\exp\left(\frac{x_{wj}^{t}-x_{ij}^{t}}{t^{2}}\right),\;i>\frac{\text{NP}}{2}\\ x_{ij}^{t+1}=x_{ij}^{t}+\left(x_{kj}^{t}-x_{ij}^{t}\right)\times \text{FL}\times \text{rand}\left(0,1\right),\;\text{otherwise} \end{array}\right). \end{array}$$

The SSA algorithm optimizes by the BSA algorithm to improve its search ability and development ability and achieves better global optimization performance. The improved ISSA algorithm optimizes SOM and applies it to the competitive selection strategy of cluster head nodes in heterogeneous sensor networks, thereby improving the efficiency of the data collection, reducing the energy consumption, and prolonging the lifetime of the network.

The sensor nodes of a heterogeneous wireless sensor network have the functions of information perception and data processing, which are similar to neurons. The sensor nodes communicate with each other and transmit data, similar to the synapses of neurons. In the cluster routing algorithm of HWSNs, when the cluster head is selected, a large number of nodes participate in the competition, but only a part of the nodes win. The learning process of the neural network is to calculate and process a large number of input vectors according to certain learning rules and finally obtain the most satisfactory solution. The two processes are very similar. Through the above similarities, the improved competitive neural network knowledge can be integrated into the routing algorithm to improve the traditional cluster head selection algorithm.

## 5. Improved Sparrow Search Algorithm Optimizes SOM in the Selection of Cluster Heads of HWSNs

### 5.1. Basic Idea of the Proposed Algorithm

The basic idea is to select the cluster head by establishing a competitive neural network, establish a mathematical model of the competitive neural network in the base station of the wireless sensor network, and use a centralized method to select the cluster head. The node at the head of the competing member is taken as the input vector, and the elements of each input vector are the remaining energy of the node, the distance from the node to the base station, and the number of neighbor nodes of the node. The node selects the most suitable head of the member through adaptive learning of the neural network. When the data are transmitted between clusters, a selection function node related to the remaining energy of the neighbor nodes of the cluster head and the distance between the cluster heads is generated during the transmission process, and the data are transferred to the neighbor node with the largest function value. The algorithm also considers setting up intermediate nodes, where the node density is small, and using these nodes to forward the data that need to be transmitted. The algorithm can effectively save and balance the energy consumption of sensor nodes and prolong the lifetime of the network.

The proposed cluster head selection strategy based on ISSA-SOM is mainly to use the principle of competitive neural network to select the best magnesium head in the cluster head selection stage. The specific implementation process is to establish a competitive neural network at the base station and select the most suitable head after the input vectors of the sensor nodes of each cluster leader are processed through the processing of the input vectors by each layer of the neural network. Consider the establishment of a selection function and intermediate nodes for data forwarding during data transmission between the cluster members. The process of this algorithm is the same as that of the algorithm, which is also divided into three stages.

The cluster routing algorithm is divided into three stages: election for cluster head, clustering, and intercluster communication. The cluster head plays a key role no matter in the cluster establishment phase or the stable operation phase between clusters. It not only collects node information in the cluster but also forwards intercluster data, so the selection of the cluster head is particularly important. This paper mainly starts from the point of view of node residual energy and node degree to avoid nodes with low energy being elected as cluster heads. The node degree is the number of neighbor nodes included in the communication range of any node in the network. In the clustering routing algorithm, in order to balance the energy consumption of the head of the member in the entire network, many researchers have studied the first stage and proposed improved algorithms. These algorithms are mainly divided into two ways to select the head of the member. This article combines neural network to select the cluster head. In this algorithm, a competitive neural network is established at the base station. At the beginning of the algorithm, each node reports its own geographic location information, remaining energy, and the number of neighbor nodes to the base station. These nodes are used as the input vector of the competitive neural network. After continuous learning, the node that is most suitable to serve as the cluster head is finally selected. After learning through the neural network, the closer the node is to the base station, the greater the remaining energy, and the number of neighbor nodes becomes the final winner. The competitive neural network proposed in this paper is divided into layers: input layer, competition layer, and output layer. Competitive neural networks are trained using competitive unsupervised learning algorithms. After the training is introduced, the best leader is selected.

In the cluster formation stage, the method used is the same as that in the traditional algorithm. After the cluster head is selected, the member head broadcasts the elected message to the surrounding nodes, and the surrounding nodes decide which the member join according to the strength of the received cluster head broadcast signal.

After the clusters are formed, the data transmission phase starts the data transmission between the clusters.

The time complexity indirectly reflects the length of time the algorithm executes. In the ISSA-SOM algorithm, it is assumed that the execution time required to initialize the parameters (under the condition that the population size is *N* and the spatial dimension is *n*) is *x*_1_, and the time to generate a uniform distribution is *x*_2_. The time required to find the fitness value is *f*(*n*); then, the time complexity of the initial stage of theSSA-SOM algorithm is as follows:

(11) $$\begin{array}{c} O\left(x_{1}+N\left(nx_{2}+f\left(n\right)\right)\right)=O\left(n+f\left(n\right)\right). \end{array}$$

Assuming that the execution time required for the iterative update of each dimension of the individual is the same, which is *x*_3_, the time for comparing the advantages and disadvantages and selecting the best after iteration is *x*_4_. Then, the time complexity of the algorithm at this stage is

(12) $$\begin{array}{c} O\left(N\left(nx_{3}+f\left(n\right)\right)+x_{4}+x_{5}+x_{6}\right)=O\left(n+f\left(n\right)\right). \end{array}$$

Therefore, the total time complexity of the SSA-SOM algorithm is

(13) $$\begin{array}{c} T\left(n\right)=O\left(n+f\left(n\right)\right)+O\left(n+f\left(n\right)\right)=O\left(n+f\left(n\right)\right). \end{array}$$

In the ISSA-SOM algorithm, the time required for the initialization phase of the algorithm is basically the same as the SSA-SOM algorithm. Therefore, the time complexity of the initialization phase of the improved algorithm is the same as equation (12). In the algorithm loop, suppose the calculation time of the weighted center is *z*_1_, the calculation time of the individual learning position is *z*_2_, and the calculation time of the comparison and selection process between the learning individual and the initial individual is *z*_3_. Then, the time complexity of the loop part is

(14) $$\begin{array}{c} O\left(N\left(nx_{3}+f\left(n\right)\right)+x_{4}+x_{5}+x_{6}+N\left(z_{2}+z_{3}\right)+z_{1}\right)=O\left(n+f\left(n\right)\right). \end{array}$$

Therefore, the total time complexity of the ISSA-SOM algorithm to solve the optimal of each generation is

(15) $$\begin{array}{c} T\left(n\right)=O\left(n+f\left(n\right)\right)+O\left(n+f\left(n\right)\right)=O\left(n+f\left(n\right)\right). \end{array}$$

In summary, the improved strategy of the ISSA-SOM algorithm does not increase the time complexity of the algorithm solution compared to the initial SSA-SOM algorithm.

### 5.2. The Work Flow of the Proposed Algorithm

Algorithm 1 shows the implementation steps of the cluster head selection strategy of HWSNs based on the improved sparrow search algorithm to optimize SOM (ISSA-SOM).

## 6. Simulation Results and Analysis

In the construction of the simulation environment, we use MATLAB 2017a software to simulate and verify the HWSNs cluster head selection strategy of the proposed ISSA-SOM algorithm. The simulated network environment is that 100 sensor nodes are randomly and evenly distributed in a 200m × 200 m square monitoring area, and all nodes will no longer move once deployed. The coordinates of the base station are the center of the monitoring area. It is assumed that the initial energy of all nodes is 0.5 J, and each round of nodes in the network is used as a data source to send data to the sink node, and the number of rounds is 2000. The algorithm parameters are set as follows: the learning rate of the SOM neural network is 0.1, and the number of learning iterations is 200. The population size of the intelligent optimization algorithm is set to 40, the dimension is 6, and the maximum number of iterations is set to 200. SSA algorithm parameter settings: the number of discoverers accounted for 20% of the entire population, the number of sparrows aware of danger SD accounted for 10%, and the safety threshold ST is 0.8. In GWO, the initial value of the distance control parameter is set to 2, and the end value of the distance control parameter is 0. Both *r*_1_ and *r*_2_ are random numbers, between [0, 1]. PSO algorithm sets learning factors *c*_1_ = 2 and *c*_2_ = 2 and inertia weight factor *ω*_1_ = 0.9 and *ω*_2_ = 0.4. The simulation parameters of the algorithm are shown in Table 1.

This paper compares the proposed algorithm with particle swarm optimization SOM (PSO-SOM), gray wolf algorithm optimization SOM (GWO-SOM), and sparrow search algorithm optimization SOM (SSA-SOM). At the same time, the number of surviving nodes, the average energy consumption, the residual energy, the number of cluster heads per round, the energy consumption of cluster heads per round, the number of packets received by sink, the network load balance, and the network connectivity and reliability are simulated.

### 6.1. Number of Surviving Sensor Nodes

**Figure 1:**
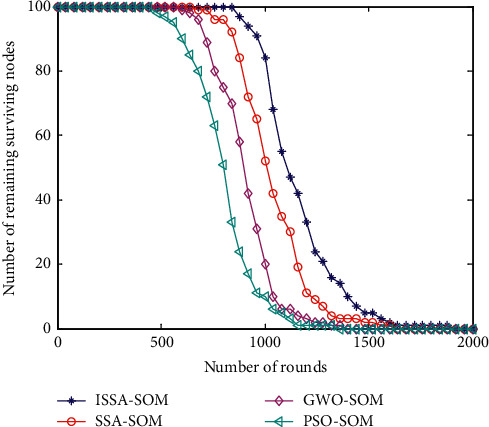
Number of surviving nodes.

The number of surviving sensor nodes is a direct manifestation of the life of a heterogeneous sensor network. The comparison of the number of the surviving sensor nodes of the four algorithms is shown in Figure 1.

Figure 1 shows the comparison of the number of surviving nodes between the algorithm in this paper and the PSO-SOM, GWO-SOM, and SSA-SOM algorithms. It can be seen from Figure 1 that the PSO-SOM algorithm starts to appear dead nodes in 500 rounds, the GWO-SOM algorithm appears in 600 rounds, the SSA-SOM algorithm appears in 700 rounds, and the ISSA-SOM algorithm proposed in this paper appears in 800 rounds. On the whole, the algorithm proposed in this paper and the PSO-SOM, GWO-SOM, and SSA-SOM algorithms all died in 1800, 1300, 1450, and 1600 rounds, respectively. Compared with the other three algorithms, the life cycle has been extended by 27.7%, 19.4%, and 11.1%.

### 6.2. Average Energy Consumption of Nodes

**Figure 2:**
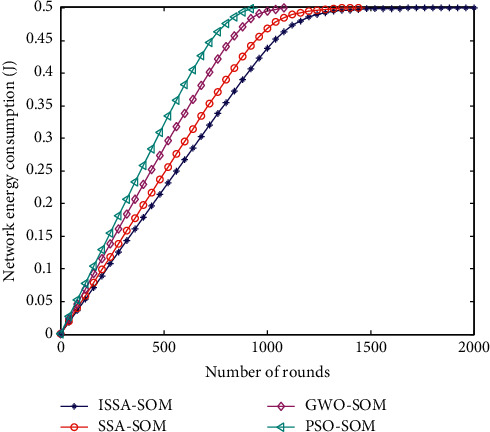
Average energy consumption of sensor nodes.

The average energy consumption of a node is one of the performance indicators of a heterogeneous wireless sensor network. The comparison of the average energy consumption of the network nodes of the four algorithms is shown in Figure 2.

Figure 2 lists the average energy consumption of nodes in the cluster head selection algorithm of four improved competitive neural networks. It can be seen from Figure 2 that starting from the algorithm simulation, the average energy efficiency of the ISSA-SOM algorithm node proposed in this paper has always been lower than that of the other three algorithms. The algorithm node energy consumption proposed in this paper is the lowest. Because the cluster head selected by this algorithm is more reasonable and the remaining energy of the neighbor cluster head and the distance between the cluster head and its neighbor cluster head are considered in the data transmission stage, and the intermediate node is forwarded; this can reduce the energy consumption during data transmission. Therefore, the average energy consumption of nodes in the proposed cluster head selection algorithm based on ISSA-SOM algorithm is lower than that of the other three algorithms.

### 6.3. Standard Deviation of Node Residual Energy

**Figure 3:**
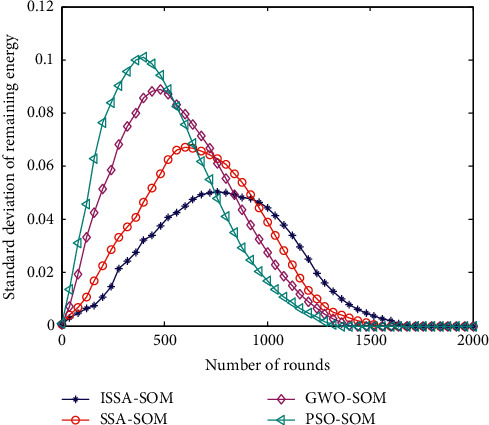
Standard deviation of node residual energy.

In order to better reflect the energy-saving performance of the proposed algorithm, we have added four algorithms to compare the remaining energy standard deviation of heterogeneous sensor nodes. The remaining energy of the node has a lot to do with the length of the network life. The standard deviation of node residual energy of the four algorithms is shown in Figure 3.

It can be seen from Figure 3 that, in the early stage of the simulation, the average energy mean square deviation of the nodes of the SSA-SOM algorithm and the ISSA-SOM algorithm proposed in this paper are both smaller than the PSO-SOM and GWO-SOM algorithms. In the later stage of the simulation, the mean square error of the node average energy of SSA-SOM algorithm and ISSA-SOM algorithm is greater than that of PSO-SOM and GWO-SOM algorithm. It shows that SSA-SOM algorithm and ISSA-SOM algorithm are better than PSO-SOM and GWO-SOM algorithm in saving network energy. This is mainly because the cluster head selected by the ISSA-SOM algorithm proposed in this paper is beneficial to balance the energy consumption of nodes.

### 6.4. Node Survival Probability

**Figure 4:**
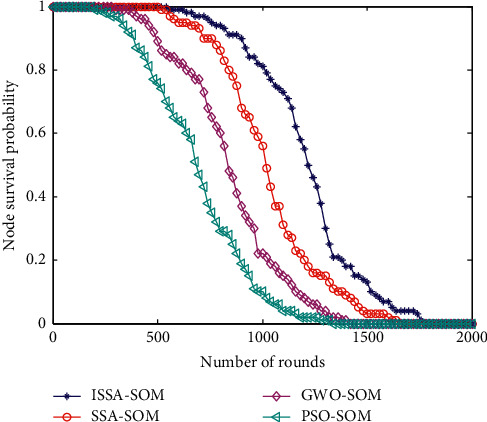
Node survival probability.

Node survival probability is also one of the most important indicators to reflect the life of the network, and many simulation indicators are used in wireless sensor networks. The comparison effect of node survival probability of the four algorithms is shown in Figure 4.

It can be seen from Figure 4 that, at the beginning of the simulation, the survival probability of sensor nodes of the four algorithms is the largest, which is 100%. As the number of simulation rounds increases, the survival probability of sensor nodes is gradually decreasing, but the decline of the four algorithms is not the same. It can be seen that the node survival probability of the PSO-SOM algorithm has the largest decline, and the node survival probability of the GWO-SOM algorithm has a greater decline. The node survival probability of the SSA-SOM algorithm has a small decline, and the node survival probability of the ISSA-SOM algorithm proposed in this paper has the smallest decline. The change in the survival probability of this node corresponds to the number of surviving nodes in Figure 1, which verifies the effectiveness of the algorithm.

### 6.5. Number of Cluster Heads

**Figure 5:**
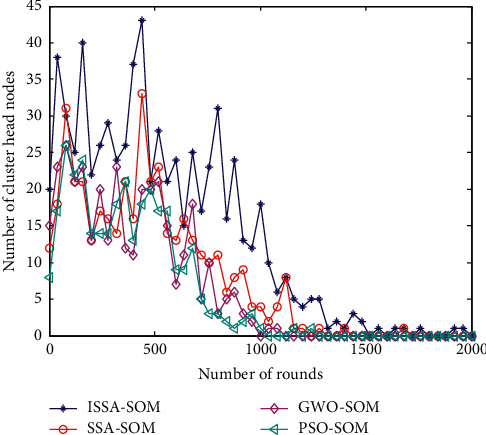
Number of cluster heads produced in each round.

The two most important indicators of the cluster head selection strategy in this paper are the number of cluster heads and the energy consumption of cluster head nodes. The number of clusters also determines the performance of the network. Too many clusters will cause frequent communication between cluster heads and sink nodes to generate greater energy consumption. If the number of clusters is too small, multihop transmission between nodes will increase energy consumption. The number of cluster heads generated by the four algorithms is shown in Figure 5.

It can be seen from Figure 5 that, as the number of simulation round increases, the number of cluster heads generated by the four algorithms remains stable. The number of cluster heads in the PSO-SOM algorithm fluctuates between 10 and 25, the number of cluster heads in the GWO-SOM algorithm fluctuates between 13 and 30, and the number of cluster heads in the SSA-SOM algorithm fluctuates between 15 and 30. This paper proposes that the number of cluster heads of ISSA-SOM algorithm fluctuates between 15 and 40. On the whole, the algorithm proposed in this paper has more cluster heads, more balanced network energy consumption, and better performance.

### 6.6. Energy Consumption of Cluster Head Nodes

**Figure 6:**
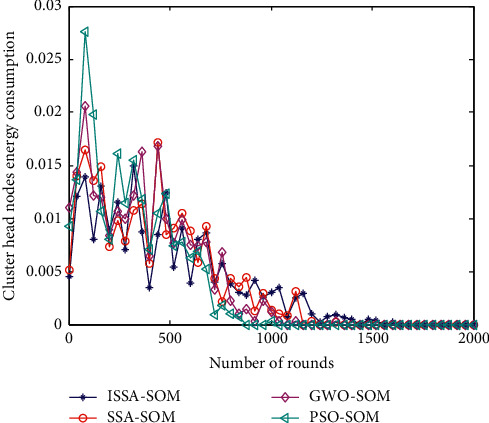
Energy consumption of cluster heads per round.

The energy consumption of the cluster head node can reflect the performance of the selected cluster head node. The lower the energy consumption, the more suitable the selected cluster head node. The energy consumption of the cluster head nodes of the four algorithms is shown in Figure 6.

It can be seen from Figure 6 that the energy consumption of the cluster head nodes in each round of the four algorithms fluctuates little. On the whole, the cluster head energy consumption of the PSO-SOM algorithm is relatively large, and the energy consumption of the cluster head nodes of the other three algorithms is not much different. The energy consumption of the cluster head node in Figure 5 is relatively large at the beginning, and the energy consumption gradually decreases as the number of rounds increases. This is mainly because as the simulation time increases, the nodes die, causing the energy consumption of the entire network to decrease.

### 6.7. Number of Packets Received by Sink

**Figure 7:**
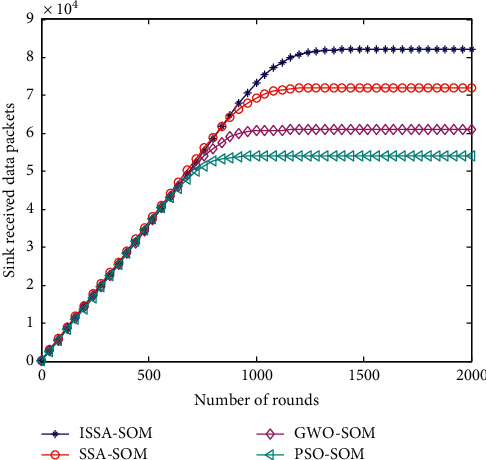
The number of packets received by Sink.

The number of packets received by the Sink is a direct manifestation of the final effect of the HWSNs' cluster head selection strategy. The more data packets are received; it indicates that the optimization strategy of the proposed cluster head selection strategy is optimal.  Figure 7 shows the comparison results of Sink nodes receiving data packets of the four algorithms.

As can be seen in Figure 6, the sink data volume of the algorithm in this paper is higher than that of the PSO-SOM, GWO-SOM, and SSA-SOM algorithms. As the simulation time increases, the amount of sink data received by the four algorithms is increasing, but the growth rate of the four algorithms is different. Among them, the sink of the PSO-SOM algorithm receives the least amount of data, the sink of the GWO-SOM algorithm receives less data, and the sink of the SSA-SOM algorithm receives more data. The sink of the ISSA-SOM algorithm proposed in this paper receives the largest amount of data. Compared with the other three, it increased by 35.7%, 27.4%, and 14.3%, respectively.

### 6.8. Network Load Balance

Wireless sensor network based on node load balancing is an important indicator for comprehensively measuring routing performance, which has important significance. The load balance analysis of the network not only ensures the reliability of data transmission in the wireless sensor network but also balances the overall load distribution of the network, prolongs the working life of the wireless sensor network, and improves the data throughput of the network at the same time. It is an important indicator of the performance parameters of wireless sensor networks. Network load balance affects energy consumption and life cycle, which is an important indicator of data collection for HWSNs [41]. The network load balancing factor of HWSNs (*L*_LBF_) refers to the inverse of the variance of all nodes in the monitoring area. The larger the value, the better the network load balance. The specific calculation formula is shown in

(16) $$\begin{array}{c} L_{\text{LBF}}=\frac{n_{c}}{\sum_{i=1}^{n_{c}}{\left(x_{i}-u\right)}^{2}}. \end{array}$$

**Figure 8:**
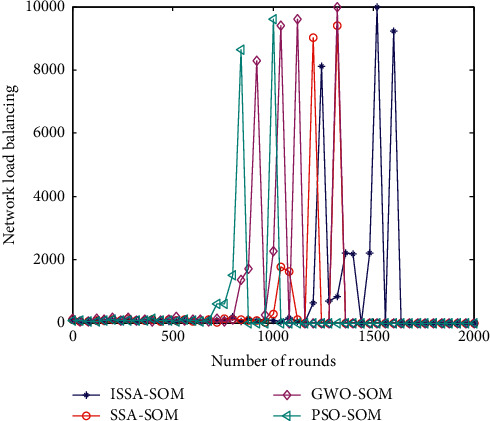
Network load balance.

The parameter *n*_*c*_ represents the total number of sensor nodes deployed in the monitoring area. The parameter *x*_*i*_ is the number of cluster member nodes added to the *i*th clustered area. The parameter *u* is the average number of cluster member nodes in all clusters. The network load balancing performance comparison of the four algorithms is shown in Figure 8.

It can be seen from Figure 8 that the network load balance has no obvious effect in the cluster head selection strategy of the four algorithms. The cluster head selection strategy balance performance of the four algorithms is general mainly because the load balance of HWSNs is mainly reflected in the data transmission between clusters, but the cluster head selection strategy in this paper is mainly for the selection of cluster heads. The network performance in network load balance is average.

### 6.9. Network Connectivity

The network connectivity of HWSNs is generally measured by the network connectivity rate, and the calculation of the connectivity rate is more complicated. The connection rate is calculated by the number of hops of data transmission, the number of hops from the source node to the destination node is counted, and the calculation of the connection rate is obtained by the data transmission traversal method of heterogeneous nodes. Taking the data transmission from heterogeneous sensor nodes of the source node to the destination node as an example, the source node transmits data to the destination node in a multihop manner until the number of nodes connected to the original source node no longer changes, that is, the network connectivity rate *N*_*c*_. The calculation formula is shown as

(17) $$\begin{array}{c} N_{c}=\frac{N_{1}}{n}. \end{array}$$

**Figure 9:**
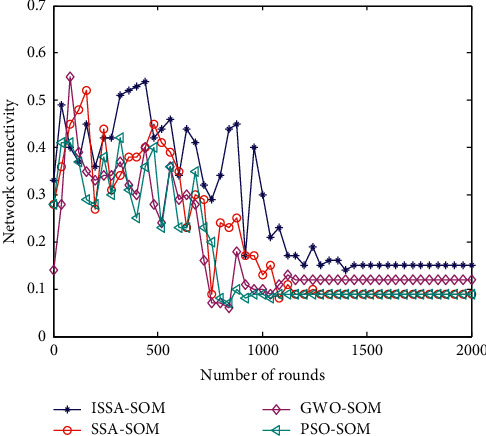
Comparison of network connectivity.

The number of all nodes that a heterogeneous sensor node can perceive is *N*_1_, and the parameter *n* is the total number of sensor nodes in the entire heterogeneous sensor network. The comparison of the HWSNs connectivity rate calculation results of the four cluster head selection strategies is shown in Figure 9.

From the comparison of the connectivity performance of the four HWSNs cluster head selection strategies in Figure 8, the network connectivity of the four algorithms all gradually decrease with the increase of the number of simulated rounds. This is mainly because, as the simulation progresses, the remaining energy of the network gradually decreases, resulting in a gradual decline in the connectivity performance of the network. From the comparison of the network connectivity performance of the four algorithms, the PSO-SOM method has the worst network connectivity. The other two cluster head selection strategies have better network connectivity. The ISSA-SOM algorithm proposed in this paper has the highest network connectivity rate and is relatively stable overall.

### 6.10. Network Reliability

**Figure 10:**
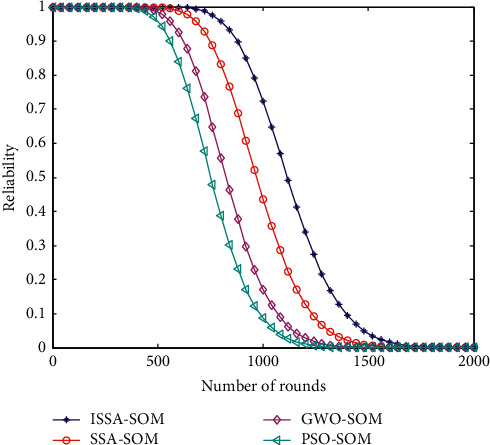
Comparison of network reliability.

Network reliability is one of the important indicators to measure the stable and normal operation of heterogeneous wireless sensor networks. The comparison of the network reliability curves of the four cluster head selection strategies is shown in Figure 10.

It can be seen from Figure 9 that, after 500 rounds, the network reliability of the PSO-SOM algorithm has dropped significantly, and the ISSA-SOM algorithm proposed in this paper has a relatively small decrease in network reliability. Taking the network reliability value of 0.8 as an example, the reliability of the PSO-SOM algorithm drops to 0.95 after 600 rounds and the GWO-SOM algorithm drops to 0.95 after 720 rounds. After 830 rounds, the reliability of the proposed ISSA-SOM algorithm drops to 0.95. After 1000 rounds, the system stability is still higher than 0.95. The proposed ISSA-SOM algorithm has the best reliability in the process of cluster head selection.

The performance indicators of simulation experiments in this paper are as follows: number of surviving sensor nodes, average energy consumption of nodes, standard deviation of node residual energy, node survival probability, number of cluster heads, energy consumption of cluster head nodes, number of packets received by Sink, network load balance, network connectivity, and network reliability. The standardization indexes of wireless sensor networks are related and affect each other. For example, the lifetime of the network is positively correlated with the number of surviving sensor nodes and negatively correlated with the average energy consumption of network sensor nodes.

In these simulation experiments, the network load balance, network connectivity, network reliability, and other indicators can better illustrate the performance of the algorithm proposed in this paper. The better the network performance of these algorithms, the higher the value, indicating that the network has the best performance, the longest service life, and the lowest energy consumption.

From the comprehensive performance evaluation of these simulation experiments, the ISSA-SOM algorithm proposed in this paper has the highest data collection efficiency, the best performance, the longest service life, and the highest network load balance, network connectivity, and reliability. From the performance indicators of these wireless sensor networks done in this paper, comprehensively, the proposed algorithm effectively reduces network energy consumption, balances network energy consumption, and prolongs the lifetime of the network.

## 7. Conclusion and Future Work

Data collection and cluster head selection for heterogeneous wireless sensor networks are very important. The proper selection of cluster head nodes can greatly improve the performance of heterogeneous wireless sensor networks. This paper proposes an improved SOM algorithm based on the sparrow search algorithm to optimize competitive neural networks, focusing on the selection stage of cluster head nodes. In the cluster head selection stage, a competitive neural network model is established at the base station, and the nodes of the competing cluster heads are used as input vectors. The vector includes three elements: the remaining energy of the node, the number of nodes, and the distance from the node to the base station. The optimal cluster head is selected through the adaptive learning of the improved SOM. The proposed algorithm greatly improves the network life cycle and data transmission volume, reduces network energy consumption, and improves network performance.

The main work of this paper is the cluster head selection strategy in the first stage of clustered data collection. The number of clusters in the second stage is also a complex optimization process, which has a great impact on the data collection efficiency of HWSNs and the life of the network. The follow-up focus will be on the number of clusters in the second stage. In the future research work, the influence of mobile nodes on the cluster head selection mechanism of heterogeneous sensor networks will be considered at the same time.

## Figures and Tables

**Algorithm 1:**
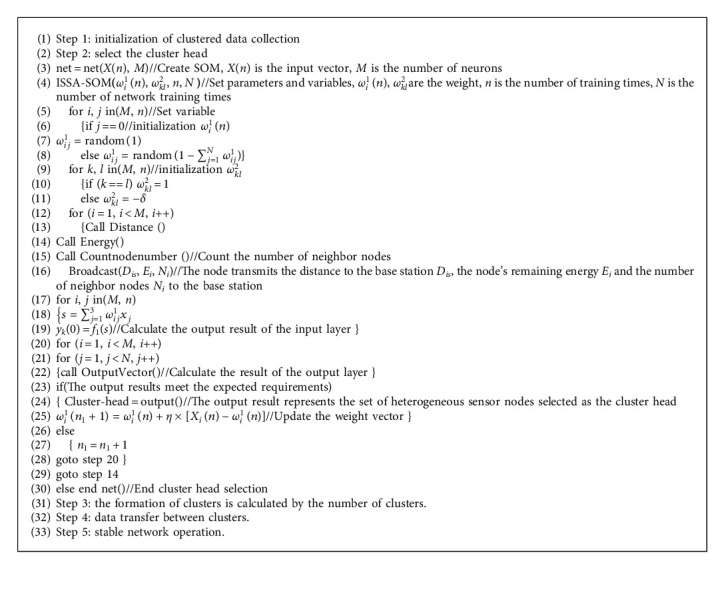
Pseudocode of cluster head selection strategy.

**Table 1:** Simulation environment parameter setting.

Parameter	Value
Network range	200 × 200 m^2^
Number of nodes	100
Communication radius	30 m
Initial energy	0.5 J
*E*_*elec*_	50 nJ/bit
*E*_*fs*_	10 pJ/bit/m^2^
*E*_*amp*_	0.0013 pJ/bit/m^4^
*l*	4000 bits
*d*_0_	$\sqrt{E_{fs}/E_{mp}}=87\text{ m}$

## Data Availability

The data used to support the findings of this study are available from the corresponding author upon request.
